# PHSP-Net: Personalized Habitat-Aware Deep Learning for Multi-Center Glioblastoma Survival Prediction Using Multiparametric MRI

**DOI:** 10.3390/bioengineering12090978

**Published:** 2025-09-15

**Authors:** Tianci Liu, Yao Zheng, Chengwei Chen, Jie Wei, Dong Huang, Yuefei Feng, Yang Liu

**Affiliations:** 1 School of Biomedical Engineering, Air Force Medical University, No. 169 Changle West Road, Xi’an 710032, China; liutianci@fmmu.edu.cn (T.L.); zhengyao0202@fmmu.edu.cn (Y.Z.); chen18631930732@163.com (C.C.); jieweiwtt@fmmu.edu.cn (J.W.); huangdong1007785@outlook.com (D.H.); fengyuefei@fmmu.edu.cn (Y.F.); 2 Innovation Reasearch Institute, Xijing Hospital, Air Force Medical University, No. 169 Changle West Road, Xi’an 710032, China; 3 Shaanxi Provincial Key Laboratory of Bioelectromagnetic Detection and Intelligent Perception, No. 169 Changle West Road, Xi’an 710032, China

**Keywords:** glioblastoma, radiomics, habitat imaging, overall survival, machine learning

## Abstract

Background: Glioblastoma (GBM) is a highly aggressive and heterogeneous primary malignancy of the central nervous system, with a median overall survival (OS) of approximately 15 months. Achieving accurate and generalizable OS prediction across multi-center settings is essential for clinical application. Methods: We propose a Personalized Habitat-aware Survival Prediction Network (PHSP-Net) that integrates multiparametric MRI with an adaptive habitat partitioning strategy. The network combines deep convolutional feature extraction and interpretable visualization modules to perform patient-specific subregional segmentation and survival prediction. A total of 1084 patients with histologically confirmed WHO grade IV GBM from four centers (UPENN-GBM, UCSF-PDGM, LUMIERE and TCGA-GBM) were included. PHSP-Net was compared with conventional radiomics, habitat imaging models and ResNet10, with independent validation on two external cohorts. Results: PHSP-Net achieved an AUROC of 0.795 (95% CI: 0.731–0.852) in the internal validation set, and 0.707 and 0.726 in the LUMIERE and TCGA-GBM external test sets, respectively—outperforming both comparison models. Kaplan–Meier analysis revealed significant OS differences between predicted high- and low-risk groups (log-rank *p* < 0.05). Visualization analysis indicated that necrotic-region habitats were key prognostic indicators. Conclusions: PHSP-Net demonstrates high predictive accuracy, robust cross-center generalization and improved interpretability in multi-center GBM cohorts. By enabling personalized habitat visualization, it offers a promising non-invasive tool for prognostic assessment and individualized clinical decision making in GBM.

## 1. Introduction

Glioblastoma (GBM) is the most common primary malignant brain tumor in adults, accounting for nearly half of all malignant gliomas and approximately 15% of all primary brain tumors, characterized by aggressive infiltration, substantial intratumoral heterogeneity and a dismal prognosis [1,2]. Although most patients undergo maximal safe surgical resection followed by temozolomide-based chemoradiotherapy, clinical outcomes remain poor, with a median OS of approximately 15 months and a 5-year survival rate below 5% [3,4,5]. Accurately predicting OS is essential for optimizing treatment strategies and guiding individualized clinical management in GBM patients. Magnetic Resonance Imaging (MRI), owing to its non-invasive nature and superior spatial resolution, serves as a cornerstone in the diagnosis, evaluation and longitudinal monitoring of GBM [6,7].

In recent years, radiomics, habitat imaging, deep convolutional neural networks (CNNs) and other machine learning methods applied to multiparametric MRI have shown considerable promise as non-invasive approaches for predicting OS in GBM patients [8,9,10,11,12,13,14,15]. However, clinical application remains limited by two principal challenges: (1) Current studies lack large-scale, multi-center training datasets and independent external validation, and few have compared different advanced models, which weakens their reliability and generalizability. (2) Although habitat imaging is based on traditional radiomics and CNNs have shown strong performance in predicting OS in GBM patients, no studies have yet combined habitat imaging with CNNs to improve OS prediction.

To address these limitations, we assembled a multi-institutional, four-center cohort comprising 1084 GBM patients and performed a comprehensive comparative analysis of several imaging-based predictive methodologies. This study framework facilitated accurate and non-invasive OS prediction, while simultaneously enhancing model reproducibility and clinical application. This study has the potential to aid clinical decision making and personalized management in GBM patients.

## 2. Materials and Methods

### 2.1. Data Sources and Description

This study utilized four publicly available datasets: (1) the University of Pennsylvania Health System glioblastoma dataset (UPENN-GBM) [16]; (2) the preoperative diffuse glioma MRI dataset from the University of California, San Francisco (UCSF-PDGM) [17]; (3) the GBM dataset from the 2021 Brain Tumor Segmentation Challenge (TCGA-GBM) [18,19]; and (4) the longitudinal GBM dataset from the University Hospital of Bern in Switzerland (LUMIERE) [20]. The UPENN-GBM, UCSF-PDGM and TCGA-GBM datasets are publicly accessible through The Cancer Imaging Archive (TCIA), supporting reproducibility and open research. Instructions for accessing the LUMIERE dataset are provided in the original publication, with corresponding access links listed in the Data Availability Statement. All datasets comprise preoperative multimodal MRI scans, three-dimensional tumor segmentation masks and associated clinical information. The UPENN-GBM dataset includes MRI scans from 630 adult patients, comprising 611 preoperative and 60 postoperative scans, collected at the University of Pennsylvania Health System. The UCSF-PDGM dataset comprises 501 histopathologically confirmed adult patients with diffuse gliomas, including 56 WHO grade II and 43 WHO grade III cases. For this study, only WHO grade IV GBM cases were retained. The TCGA-GBM dataset, which was part of the 2021 Brain Tumor Segmentation Challenge, includes preoperative MRI scans from 135 GBM patients. The LUMIERE dataset consists of longitudinal data from 91 patients with GBM.

**Figure 1:**
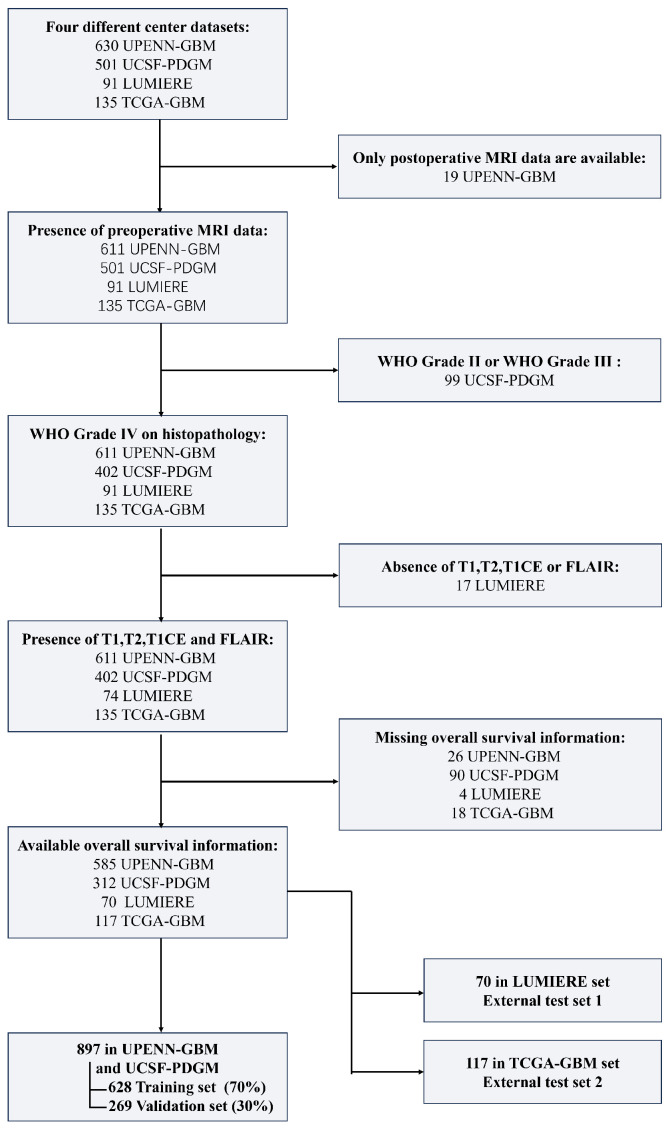
Process for inclusion of training, internal validation and two independent test sets.

**Figure 2:**
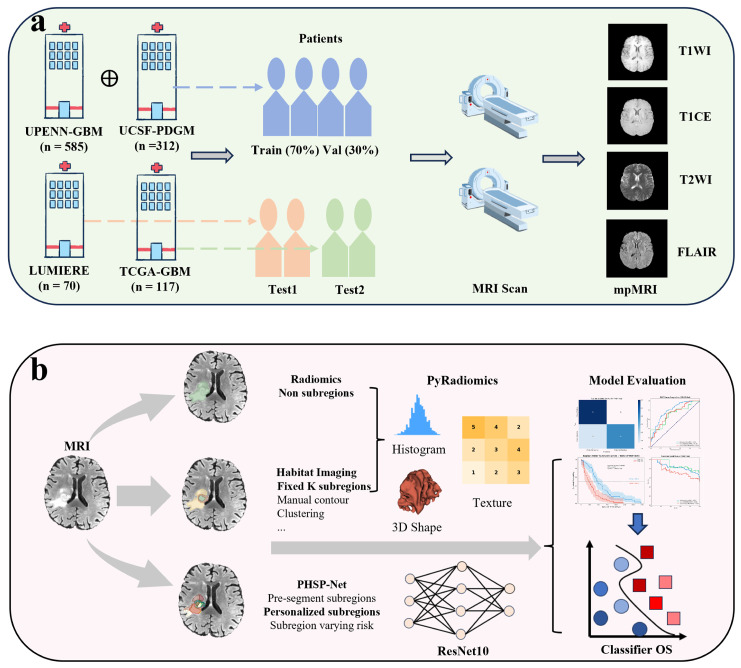
(**a**) The multi-center patient acquisition pipeline, where each patient case includes T1WI, T1CE, T2WI and FLAIR MRI sequences, along with a corresponding three-dimensional tumor mask. (**b**) Comparison of the four methodological approaches. The radiomics approach treats the tumor as a single, intact region. Habitat imaging partitions each lesion into a fixed number (K) of subregions. In contrast, PHSP-Net applies Simple Linear Iterative Clustering (SLIC) to independently segment the edema, necrotic core and enhancing tumor regions into 24 subregions each, followed by an end-to-end fusion process to enable patient-specific partitioning. ResNet10 is a high-performing convolutional neural network used to draw comparison with the PHSP-Net model’s performance.

Strict inclusion and exclusion criteria were applied across the four multi-center datasets: (1) preoperative MRI acquisition; (2) histopathological confirmation of WHO grade IV GBM; (3) availability of OS data; and (4) follow-up duration of at least one year postoperatively; (5) availability of multimodal preoperative imaging, including T1-weighted imaging (T1WI), T2-weighted imaging (T2WI), contrast-enhanced T1-weighted imaging (T1CE) and T2 fluid-attenuated inversion recovery (FLAIR) sequences, along with corresponding tumor segmentation masks. A detailed flowchart illustrating the inclusion process is presented in Figure 1. Clinical information for the included patients is provided in Supplementary Material Section S1. The overall study design is illustrated in Figure 2.

All datasets provided skull-stripped and spatially registered MRI sequences. These images were resampled to an isotropic resolution of 1 × 1 × 1 mm^3^, resulting in a final volume size of 240 × 240 × 155 voxels. Tumor segmentation masks in each dataset delineated three distinct subregions: the necrotic tumor core, contrast-enhancing tumor and peritumoral edema.

### 2.2. Radiomics and Habitat Imaging Feature Extraction

To facilitate OS prediction in GBM patients via machine learning, radiomic features were extracted from regions of interest (ROIs) in multiparametric MRI scans using the PyRadiomics package [21]. Two ROI types were defined: (1) the original tumor segmentation mask, encompassing the necrotic core, contrast-enhancing tumor and edema; and (2) a combined binary mask generated by merging these three subregions.

To comprehensively capture OS-predictive features, PyRadiomics settings were modified to extract both conventional features from original images and additional features from various image transformations. The image types included original images; squared images; square root images; logarithmic and exponential transformations; Gaussian-filtered images with σ=1.0, 1.5, 2.0, 2.5, and 3.0; wavelet-transformed images comprising eight sub-bands (LLL, LLH, LHL, LHH, HLL, HLH, HHL, HHH); and 3D local binary pattern (LBP) images with two intensity levels. In total, 20 distinct image types were utilized for feature extraction.

For each image type, 18 first-order statistical features, 24 gray-level co-occurrence matrix (GLCM) features, 16 gray-level run length matrix (GLRLM) features, 16 gray-level size zone matrix (GLSZM) features, 5 neighboring gray tone difference matrix (NGTDM) features and 14 gray-level dependence matrix (GLDM) features were extracted, yielding a total of 93 features. Additionally, 14 shape features were extracted exclusively from the original images, resulting in a total of 107 features per modality.

Thus, using the whole tumor as the ROI, each patient’s four MRI modalities yielded a total of (107 + 93 × 19) × 4 = 7496 features. When the three subregions (necrotic core, enhancing tumor, edema) were used as separate ROIs, the number of extracted features per patient increased to (107 + 93 × 19) × 4 × 3 = 22,488. The detailed feature extraction process and feature names are provided in Supplementary Material Section S2. The habitat imaging model is formulated as follows:

(1) HabitatFeature=Habitat1Feature1+Habitat2Feature2+Habitat3Feature3

where Habitat1, Habitat2 and Habitat3 represent the necrotic, enhancing and edematous regions, respectively, and Feature1, Feature2 and Feature3 correspond to the extracted features for each region.

### 2.3. Feature Selection

To reduce feature redundancy and mitigate overfitting, a multi-step feature selection process was applied, including data normalization and standardization, univariate analysis (U-test) to retain features with *p* < 0.05, Pearson correlation analysis to eliminate highly correlated features and, finally, the Least Absolute Shrinkage and Selection Operator (LASSO) [22] to select features with non-zero coefficients. Detailed methods are outlined in Supplementary Material Section S3.

### 2.4. Conventional Machine Learning Models

After selecting the features most strongly associated with OS, predictive models were developed using support vector machines (SVMs) [23]. Hyperparameters, including the regularization parameter, kernel type and kernel-specific parameters, were optimized using grid search. Models utilizing conventional radiomics features are termed “conventional radiomics models”, whereas those incorporating habitat-derived features are referred to as “habitat imaging models”. Further details are provided in Supplementary Material Section S4.

### 2.5. Personalized Habitat-Aware Survival Prediction Network

**Figure 3:**
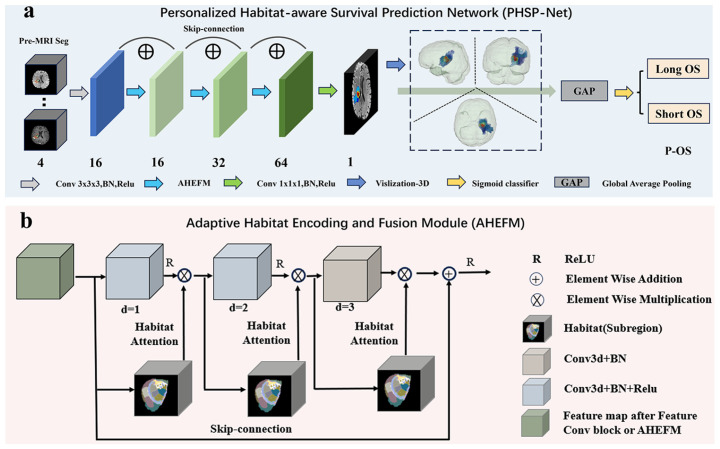
Architecture of PHSP-Net. (**a**) The overall workflow of PHSP-Net for predicting OS; (**b**) the detailed structure of the AHEFM.

We developed a novel deep learning architecture, the Personalized Habitat-aware Survival Prediction Network (PHSP-Net), to automatically partition tumor subregions (habitats) and predict OS in GBM patients. The goal is to provide a more rapid, accurate and clinically meaningful estimation of survival. PHSP-Net comprises three main modules: (1) an imaging feature extraction module; (2) a habitat output module; and (3) a global average pooling module. Within the feature extraction module, the Adaptive Habitat Encoding and Fusion Module (AHEFM) plays a key role by employing dilated convolutions to expand the receptive field while preserving high-resolution feature maps. The habitat attention mechanism recalibrates the importance of each subregion. The habitat output module visualizes the redefined subregions. PHSP-Net predicts OS and concurrently provides interpretable habitat visualizations. An overview of the network is presented in Figure 3. Detailed descriptions and formulas are available in Supplementary Materials Sections S4 and S5.

### 2.6. Model Evaluation

To enhance generalizability and prevent overfitting, the combined datasets from UPENN-GBM and UCSF-PDGM were randomly split, with 70% allocated for training and 30% for internal validation. The LUMIERE and TCGA-GBM datasets served as two independent external test sets.

OS prediction was framed as a binary classification task, aiming to predict whether a GBM patient’s survival will exceed 12 months. Evaluation metrics included accuracy, sensitivity, specificity, Area Under the Receiver Operating Characteristic curve (AUROC), Area Under the Precision–Recall Curve (AUPRC), precision and F1-score. We computed 95% confidence intervals for these metrics using 1000 bootstrap resampling iterations. Model performance was visualized by plotting ROC curves for the internal validation and external test sets. Kaplan–Meier survival curves were also generated to compare stratified patient groups predicted by different models. Finally, the 2D and 3D outputs of the PHSP-Net model were visualized to showcase the segmentation results of the personalized habitats. These results were then compared with those from both the radiomics model and the habitat imaging model. Detailed descriptions of the objective evaluation metrics and the confusion matrix results for each model are provided in Supplementary Material Section S6.

## 3. Results

### 3.1. Characteristics of GBM Patients

This study utilized four publicly available datasets, encompassing a total of 1357 GBM patients. To the best of our knowledge, this represents the study with the largest number of GBM patients to date. After applying strict exclusion criteria, a total of 1084 eligible GBM samples were included in the analysis. Among these, the training set included 627 patients with a median OS of 14.03 months, the internal validation set comprised 270 patients with a median OS of 13.68 months, the first independent external test set included 70 patients with a median OS of 17.62 months, and the second independent external test set consisted of 117 patients with a median OS of 12.32 months (details in Supplementary Material Table S1).

### 3.2. Feature Selection Analysis

**Figure 4:**
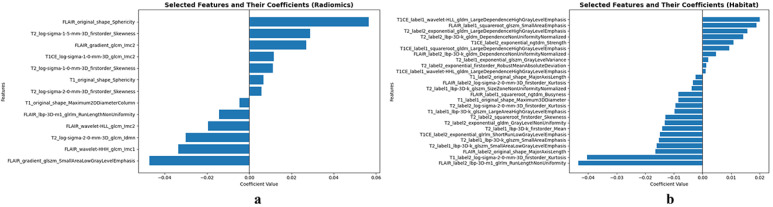
Non-zero features selected by LASSO. (**a**) Features selected from the radiomics model and (**b**) features selected from the habitat imaging model.

The extracted imaging features were first normalized and standardized. The U-test was then applied to select features significantly associated with survival (*p* < 0.05). Among the 7496 features extracted using the radiomics model, 475 features were selected, while 2130 features were selected from the 22,488 features extracted using the habitat imaging model. Next, features with a Pearson correlation coefficient greater than 0.5 were selected. The LASSO algorithm was applied with ten-fold cross-validation to select the optimal penalty parameter while simultaneously choosing features with non-zero coefficients. The optimal penalty parameter for the radiomics model was 0.00741, resulting in the selection of 13 non-zero coefficient features. For the habitat imaging model, the optimal penalty parameter was 0.01776, leading to the selection of 26 non-zero coefficient features. The names of the selected features and their non-zero coefficients are displayed in Figure 4.

### 3.3. Model Analysis

To accurately predict the OS of GBM patients, a PHSP-Net was developed, which individualizes the number of habitats for each patient. This approach was compared with traditional radiomics and habitat imaging methods. To assess the model’s predictive performance, AUROC and AUPRC curve analyses were performed. Specifically, multi-sequence MRI images (T1WI, T2WI, T1CE and FLAIR) from GBM patients were used to predict one-year OS using traditional radiomics, habitat imaging, ResNet10 and PHSP-Net deep learning methods.

**Figure 5:**
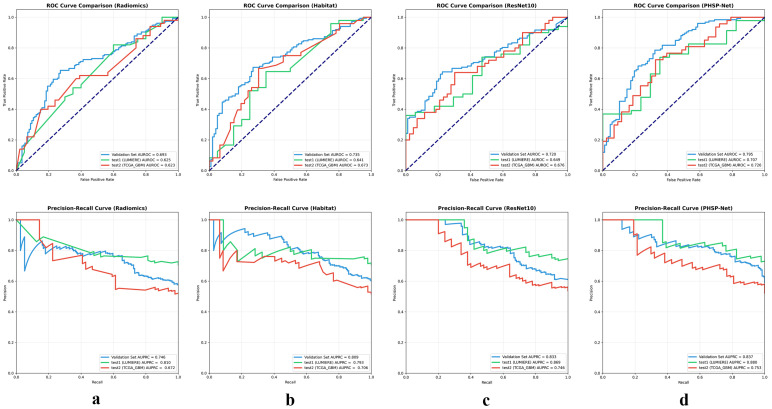
AUROC and AUPRC curves for each model. The first row represents the model-predicted AUROC curves, and the second row represents the model-predicted AUPRC curves. (**a**), (**b**), (**c**) and (**d**) correspond to the model results for radiomics, habitat imaging, ResNet10 and PHSP-Net, respectively.

The results indicated that the PHSP-Net method demonstrated the highest performance in predicting OS. Specifically, the model achieved AUROCs of 0.795 (95% CI: 0.731–0.852) in the validation set, 0.707 (95% CI: 0.632–0.779) in the first independent test set (LUMIERE) and 0.726 (95% CI: 0.649–0.797) in the second independent test set (TCGA-GBM). Compared to traditional radiomics, habitat imaging and ResNet, the AUROC of PHSP-Net increased by 10.2%, 6.0% and 7.5% in the validation set, 8.2%, 6.6% and 5.8% in the first independent test set, and 10.3%, 5.3% and 5.0% in the second independent test set, respectively. The AUROC curves are shown in the first row of Figure 5, the AUPRC curves are shown in the second row of Figure 5, and the detailed evaluation metrics for each model are presented in Table 1.

**Figure 6:**
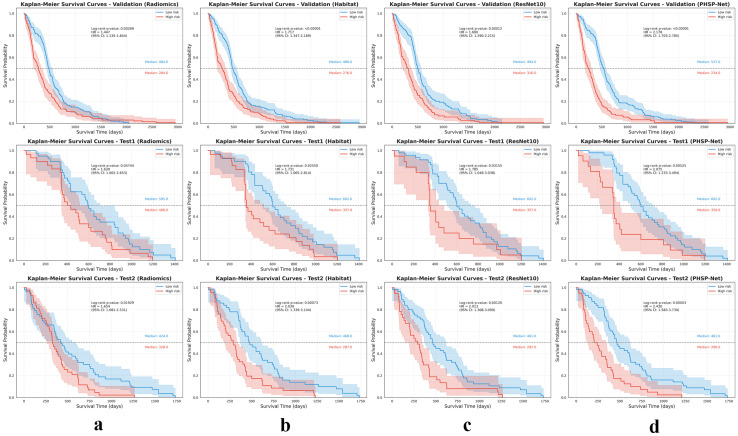
Kaplan–Meier (KM) curves for the models are presented as follows: the first, second and third rows represent the internal validation set, independent test set 1 (LUMIERE) and independent test set 2 (TCGA–GBM), respectively. (**a**), (**b**), (**c**) and (**d**) correspond to the radiomics, habitat imaging, ResNet10 and PHSP-Net models, respectively.

Furthermore, based on the predicted results from the three models (using the threshold values derived from the AUROC curve), patients were classified into low and high risk groups. Kaplan–Meier survival curves demonstrated significant differences in survival between the groups, confirmed by the log-rank test, further validating the model’s effectiveness in predicting OS, as shown in Figure 6.

### 3.4. Model Visualization

**Figure 7:**
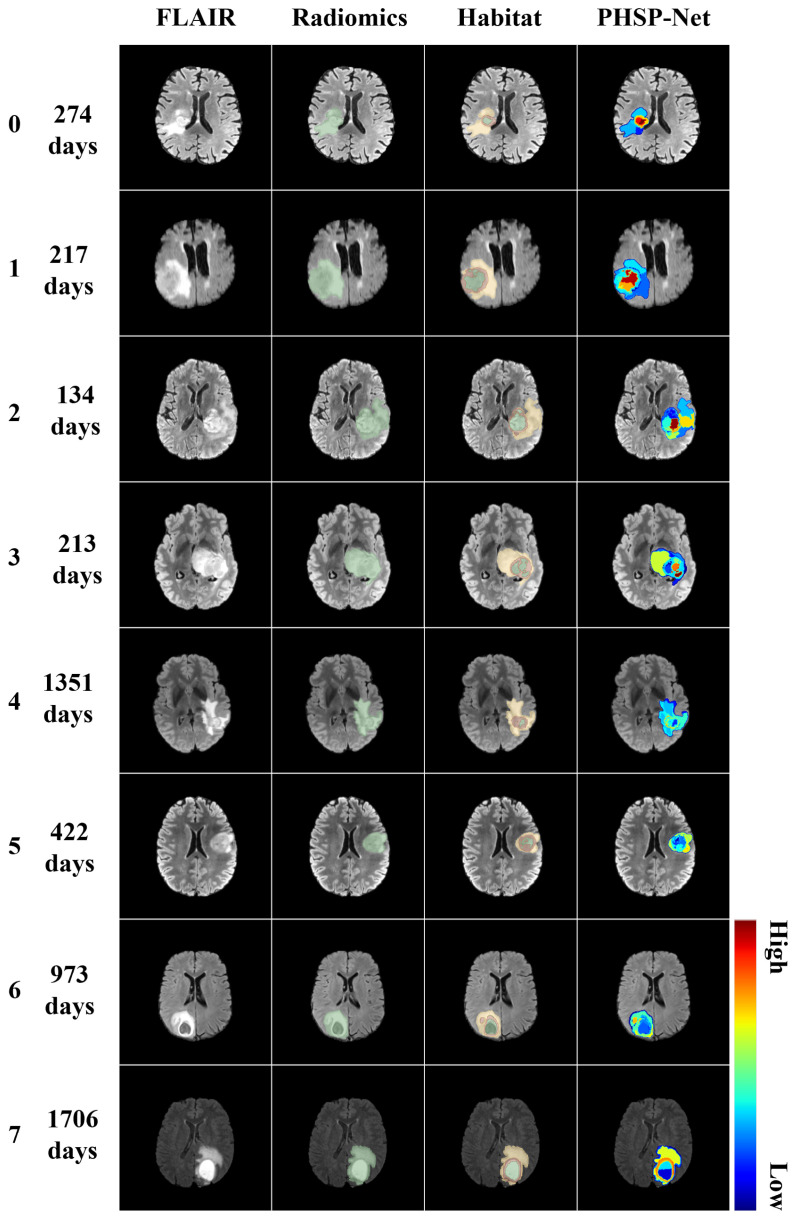
From left to right, the figure shows the FLAIR image, followed by the 2D visualizations of the ROIs for the radiomics, habitat imaging and PHSP-Net models. A color jitter scheme was applied to visually represent the risk of OS across different habitat and radiomics features in patients. Red tones denote higher risk. Patients with survival less than one year are labeled with values 0–3, whereas those with survival times exceeding one year are labeled with values 4–7. Each number from 0 to 7 corresponds to a patient, along with their specific OS time.

**Figure 8:**
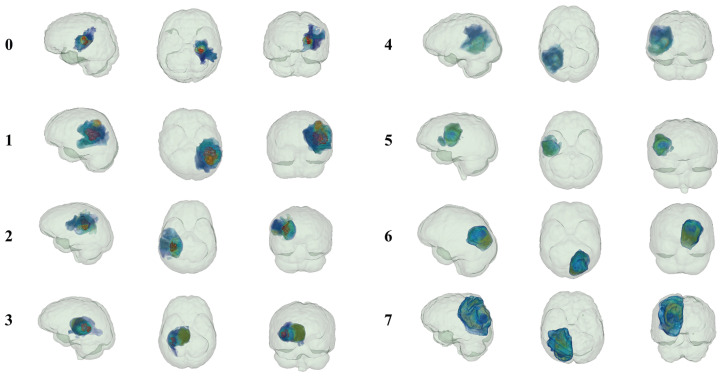
The PHSP-Net-derived 3D habitat maps correspond to the 2D patient visualizations shown above, providing a comprehensive spatial representation of the habitat regions delineated by PHSP-Net in three-dimensional space. Consistent with Figure 7, the numbers 0–7 correspond to the same patients.

In contrast to traditional radiomics and habitat imaging, PHSP-Net personalizes the number of habitats for each patient, offering potential assistance with clinical tasks such as diagnosis, disease progression management and clinical decision making. Unlike traditional radiomics and habitat imaging methods, PHSP-Net provides personalized habitat segmentation for each patient, which can potentially aid in disease diagnosis, management and clinical decision making. In this study, four long-term survivors and four short-term survivors were randomly selected from the test cohort for both 2D and 3D visualization. PHSP-Net heat maps demonstrated that necrotic regions were highlighted in short-term survivors, whereas they received less attention from the model in patients with prolonged survival. The PHSP-Net visualization results not only characterize the risk comparison of different habitats within the same patient but also depict risk profiles across different patients and habitats, providing clinicians with valuable insights for assessing patient prognosis. The results of personalized habitat segmentation and the 2D visualizations for both the traditional radiomics and habitat imaging methods are shown in Figure 7, while the 3D visualization results of personalized habitat segmentation by the PHSP-Net model are displayed in Figure 8.

## 4. Discussion

Accurate, non-invasive prediction of OS in the highly heterogeneous context of glioblastoma remains a significant challenge in imaging science and clinical oncology [1,24]. Developing machine learning models that not only achieve precise OS estimation but also demonstrate robust generalization across different clinical centers is a pressing unmet need. Reliable survival prediction holds great promise for guiding clinical decision making regarding surgical intervention, adjuvant chemoradiotherapy and ongoing patient management [9,15].

Non-invasive prediction of OS in GBM has advanced rapidly through machine learning approaches [8,9,10,11,12,13,14,15]; however, these promising results have almost universally been derived from small, single-center cohorts, limiting their generalizability to real-world clinical practice. For instance, Xin et al. [25] achieved an AUROC of 0.919 in a validation set of 38 GBM patients, and Xu et al. [26] reported an accuracy of 0.875 in an external cohort of 30 cases. Despite these impressive metrics, the lack of large-scale, multi-institutional validation has hindered translation into real-world clinical practice. The present study addresses this gap by training and independently validating models on a dataset comprising over one thousand GBM patients from four centers—the largest volume reported to date—thereby mitigating heterogeneity introduced by variable acquisition parameters and geographic case mix. Furthermore, we provide the first systematic comparison of three non-invasive paradigms—conventional radiomics, habitat imaging and personalized habitat-based deep learning—for 12-month OS classification, substantially enhancing the reproducibility and generalizability of our findings.

Conventional radiomics treats the entire tumor as a homogeneous region of interest and extracts high-throughput features from the whole tumor. Such an approach inevitably overlooks GBM’s marked spatial heterogeneity and fails to capture subtle subregional signals that may be prognostically decisive, thereby limiting incremental gains in predictive accuracy [9,27]. Habitat imaging attempts to overcome this limitation by partitioning the tumor into a predefined number of subregions, or habitats, via unsupervised clustering or manual segmentation, followed by feature extraction within each compartment [28,29]. Although this approach enhances the radiomics signature, choosing the habitat count beforehand introduces methodological uncertainty. It remains unclear whether all patients should be assigned the same number of habitats, or which criteria should guide patient-specific partitioning. Deep learning offers an end-to-end alternative that learns task-relevant imaging patterns without explicit subregional definitions; however, its “black box” nature obscures the biological rationale underlying each prediction, deterring clinical adoption [30,31,32]. PHSP-Net, proposed in this study, combines the ability of habitat imaging to capture local image features with the automatic extraction of OS-related imaging characteristics enabled by deep learning. Using an end-to-end deep learning framework, PHSP-Net can adaptively partition and visualize a variable number of habitats for each GBM patient, thereby mitigating the "black box" nature of deep learning. Compared to conventional radiomics and habitat imaging methods, this approach demonstrates significant advantages in model performance.

Contemporary deep learning models for GBM survival prediction have surpassed traditional clinical variables in discriminative capacity [13,15,33,34,35], yet physician acceptance hinges on whether the provided explanations align with established cognitive schemas and workflow patterns [36,37,38]. Rasheed et al. [39] argue that the principal barrier to clinical deployment is not algorithmic accuracy but rather the opacity of “black box” outputs; high AUROC values consequently fail to translate into altered real-world management decisions. Salahuddin et al. [37] further emphasize that radiologists require a visual language congruent with their daily interpretive experience, and that attention mapping serves as the conduit through which model salience is projected onto native image space, thereby synchronizing machine inference with human visual reasoning.

PHSP-Net’s high-resolution 2D and 3D heat maps delineate prognostically critical habitats in a format immediately intelligible to clinicians, enabling instantaneous cross-validation between algorithmic risk markers and radiologically observed spatial anomalies [40,41,42]. Such an interpretative paradigm, akin to the “what-you-see-is-what-you-get” principle, reduces cognitive load under uncertainty and provides a tangible imaging substrate open to discussion and scrutiny during multidisciplinary tumor boards, thereby facilitating shared decision making [43,44,45]. Transparent visual explanation is therefore not only a regulatory necessity but also a pivotal step toward re-engineering GBM care pathways and achieving truly individualized medicine.

In addition to these visualization-based insights, analysis of the confusion matrices provides further perspective on model behavior (Supplementary Figure S2). Across the validation and two external test cohorts, most false predictions arose from borderline cases where the model assigned patients to the opposite risk category. Specifically, false positives corresponded to patients predicted as high-risk but who in fact survived longer than expected, while false negatives represented patients predicted as low-risk but who experienced shorter survival. Although these errors reduced overall recall (average 0.706 across validation, Test 1 and Test 2), precision remained relatively high (average 0.766 across cohorts), suggesting that when the model predicts a patient as high-risk, the classification is generally reliable. These findings indicate that the misclassifications are not random but reflect the inherent difficulty of distinguishing outcome extremes in heterogeneous populations.

In our study, PHSP-Net achieved an AUROC of 0.795 in the internal validation cohort, yet the performance decreased to 0.707 and 0.726 in the two external cohorts. This decline highlights the challenges of generalization in multi-center studies, where variability in scanner hardware, acquisition protocols and patient demographics may introduce distributional shifts. Moreover, the inherent heterogeneity of glioblastoma, together with differences in follow-up duration and clinical outcome distributions, may have contributed to the reduced discriminative capacity. These factors together explain why the external AUROC values, although superior to those of baseline models, remain only modest in magnitude.

Although overall survival in glioblastoma remains limited, preoperative prognosis estimation carries important clinical implications. Beyond statistical stratification, PHSP-Net’s ability to non-invasively identify high- and low-risk groups before any treatment is initiated provides actionable insights for patient counseling, treatment triage and enrollment into clinical trials. Patients predicted to have poor prognosis may be prioritized for escalated, experimental or palliative approaches, while those with more favorable survival likelihood could benefit from standard regimens or organ-preserving strategies [46,47]. Unlike traditional radiomics that overlook tumor heterogeneity, our habitat-aware framework highlights biologically meaningful subregions, particularly necrotic habitats, and thus offers an interpretable link between imaging phenotypes and disease aggressiveness. This individualized and visualizable prognostic information represents a key advantage of PHSP-Net, positioning it as a clinically relevant tool that bridges preoperative imaging with personalized treatment planning.

Nonetheless, translating such prognostic insights into routine practice requires consideration of practical deployment barriers. While PHSP-Net provides interpretable outputs that align with radiological reasoning, practical barriers to clinical adoption must be acknowledged. Inference time, although relatively short in research settings, may still present challenges for seamless use in high-volume clinical workflows. Moreover, integration with existing PACS systems requires standardized pipelines that ensure compatibility and minimal disruption to routine imaging practices. Finally, regulatory approval processes remain an essential step before translation into clinical deployment, underscoring the gap between algorithmic performance and real-world implementation.

In addition to these translational hurdles, several methodological limitations of the present study should be acknowledged. First, the current analysis did not incorporate advanced functional sequences, such as perfusion- or diffusion-weighted imaging. From a pragmatic standpoint, conventional multiparametric MRI remains the most universally available and protocol-complete dataset across global healthcare tiers, while DWI and DSC-PWI suffer from highly variable acquisition parameters and elevated missingness rates, which could compromise external validity. Second, non-public datasets were not utilized, as they often lack complete follow-up or requisite sequence completeness, thereby failing to meet multi-center harmonization standards. Third, this study focused on MRI data and did not incorporate prior CT scans, which, despite lower soft-tissue contrast, are often the initial imaging modality in clinical practice due to wider availability and faster acquisition, and could provide valuable a priori anatomical context in a significant number of cases. Fourth, in this study, we adopted a binary framework for overall survival prediction, which inevitably simplifies the heterogeneity of patient outcomes. Moving forward, we plan to explore more flexible modeling strategies, such as continuous survival regression or multi-level risk grouping, to better capture prognostic variability and enhance clinical relevance. Fifth, the prediction model did not incorporate genetic-molecular data (e.g., IDH mutation, MGMT promoter methylation status), which are established prognostic biomarkers in neuro-oncology. While the study’s aim was to develop a purely imaging-driven predictor, the absence of this correlative analysis limits its direct clinical translation and comparative value against the current standard of care. As a future research direction, it would be valuable to investigate potential correlations between PHSP-Net–derived prognostic risk groups and established radiological frameworks such as the Brain Tumor Reporting and Data System (BT-RADS) [48]. Since BT-RADS is increasingly adopted in clinical practice for the standardized follow-up of glioblastoma patients, aligning our imaging-based prognostic model with this classification could enhance interpretability and facilitate clinical translation.

## 5. Conclusions

This study demonstrates that PHSP-Net accurately predicts overall survival in GBM patients across a multi-center, large-scale dataset and compares the model’s performance with traditional methods, as well as testing its generalization across different centers. The results show that PHSP-Net outperforms other models and not only predicts patient survival but also provides personalized delineation of different habitat risk regions. The model’s inherent interpretability may also contribute to biological insights and guide personalized treatment decisions. We anticipate that PHSP-Net could become a highly reproducible and clinically practical tool for non-invasive prognosis assessment in GBM patients.

## Figures and Tables

**Table 1:** Performance metrics of models (95% CI). Test 1 denotes the LUMIERE test set, and Test set 2 denotes the TCGA-GBM test set.

Model	Cohort	Performance Metrics (95% CI)						
		Accuracy	Precision	Specificity	F1-Score	AUROC	AUPRC
	Validation	0.670 (0.618–0.731)	0.738 (0.664–0.811)	0.670 (0.618–0.731)	0.675 (0.585–0.761)	0.700 (0.641–0.760)	0.693 (0.629–0.756)	0.746 (0.667–0.825)
Radiomics	Test set 1 (LUMIERE)	0.571 (0.457–0.692)	0.778 (0.687–0.859)	0.571 (0.457–0.692)	0.600 (0.480–0.720)	0.651 (0.542–0.757)	0.625 (0.483–0.770)	0.810 (0.719–0.905)
	Test set 2 (TCGA-GBM)	0.573 (0.469–0.667)	0.585 (0.475–0.702)	0.573 (0.469–0.667)	0.518 (0.383–0.667)	0.603 (0.519–0.698)	0.623 (0.506–0.730)	0.672 (0.583–0.767)
	Validation	0.681 (0.624–0.740)	0.759 (0.688–0.831)	0.681 (0.624–0.740)	0.667 (0.574–0.758)	0.715 (0.664–0.779)	0.735 (0.672–0.792)	0.809 (0.737–0.872)
Habitat Imaging	Test set 1 (LUMIERE)	0.643 (0.529–0.765)	0.816 (0.702–0.909)	0.643 (0.529–0.765)	0.650 (0.565–0.742)	0.719 (0.595–0.824)	0.641 (0.530–0.758)	0.793 (0.695–0.887)
	Test set 2 (TCGA-GBM)	0.675 (0.575–0.766)	0.681 (0.566–0.808)	0.675 (0.575–0.766)	0.679 (0.574–0.759)	0.683 (0.558–0.774)	0.673 (0.558–0.784)	0.706 (0.625–0.783)
	Validation	0.685 (0.625–0.745)	0.759 (0.702–0.815)	0.667 (0.582–0.745)	0.711 (0.613–0.802)	0.710 (0.655–0.780)	0.720 (0.652–0.786)	0.833 (0.784–0.882)
ResNet10	Test set 1 (LUMIERE)	0.646 (0.522–0.689)	0.791 (0.684–0.896)	0.680 (0.551–0.804)	0.550 (0.412–0.689)	0.731 (0.622–0.828)	0.649 (0.513–0.778)	0.869 (0.802–0.937)
	Test set 2 (TCGA-GBM)	0.607 (0.516–0.697)	0.615 (0.509–0.720)	0.656 (0.526–0.782)	0.554 (0.424–0.685)	0.635 (0.543–0.726)	0.676 (0.566–0.787)	0.746 (0.653–0.837)
	Validation	0.719 (0.662–0.776)	0.780 (0.705–0.853)	0.719 (0.662–0.776)	0.702 (0.607–0.797)	0.750 (0.694–0.806)	0.795 (0.731–0.852)	0.837 (0.785–0.883)
PHSP–Net	Test set 1 (LUMIERE)	0.714 (0.603–0.825)	0.833 (0.760–0.903)	0.714 (0.603–0.825)	0.600 (0.483–0.727)	0.792 (0.698–0.886)	0.707 (0.632–0.779)	0.880 (0.812–0.945)
	Test set 2 (TCGA-GBM)	0.684 (0.600–0.768)	0.686 (0.563–0.804)	0.684 (0.600–0.768)	0.625 (0.530–0.720)	0.709 (0.632–0.786)	0.726 (0.649–0.797)	0.753 (0.673–0.828)

## Data Availability

All datasets are publicly available and have been cited. The UPENN-GBM, UCSF-PDGM and TCGA-GBM datasets are from the Cancer Imaging Archive and are used with permission. The LUMIERE dataset originates from the University Hospital of Bern, Switzerland. Here are the specific download links for each dataset: UPENN-GBM: https://www.cancerimagingarchive.net/collection/upenn-gbm/ (accessed on 23 April 2025), UCSF-PDGM: https://www.cancerimagingarchive.net/collection/ucsf-pdgm/ (accessed on 23 April 2025), LUMIERE: https://springernature.figshare.com/articles/dataset/LUMIERE_dataset_-_MRI_data_and_automated_segmentations/21249516?backTo=%2Fcollections%2FThe_LUMIERE_Dataset_Longitudinal_Glioblastoma_MRI_with_Expert_RANO_Evaluation%2F5904905&file=38249697 (accessed on 23 April 2025), and TCGA-GBM: https://www.cancerimagingarchive.net/analysis-result/rsna-asnr-miccai-brats-2021/ (accessed on 23 April 2025).

## Supplementary Materials

The following supporting information can be downloaded at https://www.mdpi.com/article/10.3390/bioengineering12090978/s1: Figure S1: LASSO Selects Features with Non-zero Coefficients; Figure S2: Confusion matrix results for different models on the validation and test sets; Figure S3: Bar charts of objective evaluation metrics for each model on the validation set and test sets. Table S1: Patient Characteristics of the Centers; Table S2: Statistical tests for clinical characteristics of patients from different centers; Table S3: List of radiomic features; Table S4: DeLong test results for AUROC comparison among models. References [49,50,51,52,53,54] are cited in the Supplementary Materials.

## Abbreviations

The following abbreviations are used in this manuscript:

GBM	Glioblastoma
OS	Overall Survival
PHSP-Net	Personalized Habitat-Aware Survival Prediction Network
MRI	Magnetic Resonance Imaging
CNNs	Convolutional Neural Networks
AUROC	Area Under the Receiver Operating Characteristic curve
AUPRC	Area Under the Precision–Recall Curve

## References

[1] Jacob F., Salinas R.D., Zhang D.Y., Nguyen P.T., Schnoll J.G., Wong S.Z.H., Thokala R., Sheikh S., Saxena D., Prokop S. (2020). A patient-derived glioblastoma organoid model and biobank recapitulates inter-and intra-tumoral heterogeneity. Cell.

[2] Van den Bent M.J., Geurts M., French P.J., Smits M., Capper D., Bromberg J.E., Chang S.M. (2023). Primary brain tumours in adults. Lancet.

[3] Verdugo E., Puerto I., Medina M.Á. (2022). An update on the molecular biology of glioblastoma, with clinical implications and progress in its treatment. Cancer Commun..

[4] Molinaro A.M., Taylor J.W., Wiencke J.K., Wrensch M.R. (2019). Genetic and molecular epidemiology of adult diffuse glioma. Nat. Rev. Neurol..

[5] Vigneswaran K., Neill S., Hadjipanayis C.G. (2015). Beyond the World Health Organization grading of infiltrating gliomas: Advances in the molecular genetics of glioma classification. Ann. Transl. Med..

[6] Haller S., Haacke E.M., Thurnher M.M., Barkhof F. (2021). Susceptibility-weighted imaging: Technical essentials and clinical neurologic applications. Radiology.

[7] Keall P.J., Brighi C., Glide-Hurst C., Liney G., Liu P.Z., Lydiard S., Paganelli C., Pham T., Shan S., Tree A.C. (2022). Integrated MRI-guided radiotherapy—Opportunities and challenges. Nat. Rev. Clin. Oncol..

[8] Le V.H., Minh T.N.T., Kha Q.H., Le N.Q.K. (2023). A transfer learning approach on MRI-based radiomics signature for overall survival prediction of low-grade and high-grade gliomas. Med. Biol. Eng. Comput..

[9] Duman A., Sun X., Thomas S., Powell J.R., Spezi E. (2024). Reproducible and interpretable machine learning-based radiomic analysis for overall survival prediction in glioblastoma multiforme. Cancers.

[10] Wang H. (2025). Multimodal MRI radiomics based on habitat subregions of the tumor microenvironment for predicting risk stratification in glioblastoma. PLoS ONE.

[11] Macyszyn L., Akbari H., Pisapia J.M., Da X., Attiah M., Pigrish V., Bi Y., Pal S., Davuluri R.V., Roccograndi L. (2015). Imaging patterns predict patient survival and molecular subtype in glioblastoma via machine learning techniques. Neuro-Oncology.

[12] Chen H., Liu Y., Pan X., Yang Q., Qiang Y., Qi X.S. (2023). A Subregion-based survival prediction framework for GBM via multi-sequence MRI space optimization and clustering-based feature bundling and construction. Phys. Med. Biol..

[13] Yang Z., Zamarud A., Marianayagam N.J., Park D.J., Yener U., Soltys S.G., Chang S.D., Meola A., Jiang H., Lu W. (2025). Deep learning-based overall survival prediction in patients with glioblastoma: An automatic end-to-end workflow using pre-resection basic structural multiparametric MRIs. Comput. Biol. Med..

[14] Ben Ahmed K., Hall L.O., Goldgof D.B., Gatenby R. (2022). Ensembles of convolutional neural networks for survival time estimation of high-grade glioma patients from multimodal MRI. Diagnostics.

[15] Tang Z., Xu Y., Jin L., Aibaidula A., Lu J., Jiao Z., Wu J., Zhang H., Shen D. (2020). Deep learning of imaging phenotype and genotype for predicting overall survival time of glioblastoma patients. IEEE Trans. Med. Imaging.

[16] Bakas S., Sako C., Akbari H., Bilello M., Sotiras A., Shukla G., Rudie J.D., Santamaría N.F., Kazerooni A.F., Pati S. (2022). The University of Pennsylvania glioblastoma (UPenn-GBM) cohort: Advanced MRI, clinical, genomics, & radiomics. Sci. Data.

[17] Calabrese E., Villanueva-Meyer J.E., Rudie J.D., Rauschecker A.M., Baid U., Bakas S., Cha S., Mongan J.T., Hess C.P. (2022). The University of California San Francisco preoperative diffuse glioma MRI dataset. Radiol. Artif. Intell..

[18] Bakas S., Reyes M., Jakab A., Bauer S., Rempfler M., Crimi A., Shinohara R.T., Berger C., Ha S.M., Rozycki M. (2018). Identifying the best machine learning algorithms for brain tumor segmentation, progression assessment, and overall survival prediction in the BRATS challenge. arXiv.

[19] Baid U., Ghodasara S., Mohan S., Bilello M., Calabrese E., Colak E., Farahani K., Kalpathy-Cramer J., Kitamura F.C., Pati S. (2021). The rsna-asnr-miccai brats 2021 benchmark on brain tumor segmentation and radiogenomic classification. arXiv.

[20] Suter Y., Knecht U., Valenzuela W., Notter M., Hewer E., Schucht P., Wiest R., Reyes M. (2022). The LUMIERE dataset: Longitudinal Glioblastoma MRI with expert RANO evaluation. Sci. Data.

[21] Lambin P., Rios-Velazquez E., Leijenaar R., Carvalho S., Van Stiphout R.G., Granton P., Zegers C.M., Gillies R., Boellard R., Dekker A. (2012). Radiomics: Extracting more information from medical images using advanced feature analysis. Eur. J. Cancer.

[22] Tibshirani R. (1996). Regression shrinkage and selection via the lasso. J. R. Stat. Soc. Ser. B Stat. Methodol..

[23] Cortes C., Vapnik V. (1995). Support-vector networks. Mach. Learn..

[24] Hajianfar G., Haddadi Avval A., Hosseini S.A., Nazari M., Oveisi M., Shiri I., Zaidi H. (2023). Time-to-event overall survival prediction in glioblastoma multiforme patients using magnetic resonance imaging radiomics. La Radiol. Medica.

[25] Jia X., Zhai Y., Song D., Wang Y., Wei S., Yang F., Wei X. (2022). A multiparametric MRI-based radiomics nomogram for preoperative prediction of survival stratification in glioblastoma patients with standard treatment. Front. Oncol..

[26] Xu Y., He X., Li Y., Pang P., Shu Z., Gong X. (2021). The nomogram of MRI-based radiomics with complementary visual features by machine learning improves stratification of glioblastoma patients: A multicenter study. J. Magn. Reson. Imaging.

[27] McGranahan N., Swanton C. (2017). Clonal heterogeneity and tumor evolution: Past, present, and the future. Cell.

[28] Gatenby R.A., Grove O., Gillies R.J. (2013). Quantitative imaging in cancer evolution and ecology. Radiology.

[29] Zhang X., Lu D., Gao P., Tian Q., Lu H., Xu X., He X., Liu Y. (2020). Survival-relevant high-risk subregion identification for glioblastoma patients: The MRI-based multiple instance learning approach. Eur. Radiol..

[30] Patrício C., Neves J.C., Teixeira L.F. (2023). Explainable deep learning methods in medical image classification: A survey. ACM Comput. Surv..

[31] Cai L., Fang H., Xu N., Ren B. (2024). Counterfactual causal-effect intervention for interpretable medical visual question answering. IEEE Trans. Med. Imaging.

[32] Wu X., Zhang Y.T., Lai K.W., Yang M.Z., Yang G.L., Wang H.H. (2024). A novel centralized federated deep fuzzy neural network with multi-objectives neural architecture search for epistatic detection. IEEE Trans. Fuzzy Syst..

[33] Luckett P.H., Olufawo M., Lamichhane B., Park K.Y., Dierker D., Verastegui G.T., Yang P., Kim A.H., Chheda M.G., Snyder A.Z. (2023). Predicting survival in glioblastoma with multimodal neuroimaging and machine learning. J. Neuro-Oncol..

[34] Kaur G., Rana P.S., Arora V. (2023). Deep learning and machine learning-based early survival predictions of glioblastoma patients using pre-operative three-dimensional brain magnetic resonance imaging modalities. Int. J. Imaging Syst. Technol..

[35] Zheng Y., Carrillo-Perez F., Pizurica M., Heiland D.H., Gevaert O. (2023). Spatial cellular architecture predicts prognosis in glioblastoma. Nat. Commun..

[36] Martin S.A., Townend F.J., Barkhof F., Cole J.H. (2023). Interpretable machine learning for dementia: A systematic review. Alzheimer’s Dement..

[37] Salahuddin Z., Woodruff H.C., Chatterjee A., Lambin P. (2022). Transparency of deep neural networks for medical image analysis: A review of interpretability methods. Comput. Biol. Med..

[38] Zhang Y.P., Zhang X.Y., Cheng Y.T., Li B., Teng X.Z., Zhang J., Lam S., Zhou T., Ma Z.R., Sheng J.B. (2023). Artificial intelligence-driven radiomics study in cancer: The role of feature engineering and modeling. Mil. Med. Res..

[39] Rasheed K., Qayyum A., Ghaly M., Al-Fuqaha A., Razi A., Qadir J. (2022). Explainable, trustworthy, and ethical machine learning for healthcare: A survey. Comput. Biol. Med..

[40] Camalan S., Mahmood H., Binol H., Araujo A.L.D., Santos-Silva A.R., Vargas P.A., Lopes M.A., Khurram S.A., Gurcan M.N. (2021). Convolutional neural network-based clinical predictors of oral dysplasia: Class activation map analysis of deep learning results. Cancers.

[41] Ghorbani A., Ouyang D., Abid A., He B., Chen J.H., Harrington R.A., Liang D.H., Ashley E.A., Zou J.Y. (2020). Deep learning interpretation of echocardiograms. NPJ Digit. Med..

[42] Izadyyazdanabadi M., Belykh E., Cavallo C., Zhao X., Gandhi S., Moreira L.B., Eschbacher J., Nakaji P., Preul M.C., Yang Y. (2018). Weakly-supervised learning-based feature localization for confocal laser endomicroscopy glioma images. Proceedings of the International Conference on Medical Image Computing and Computer-Assisted Intervention.

[43] Kermany D.S., Goldbaum M., Cai W., Valentim C.C., Liang H., Baxter S.L., McKeown A., Yang G., Wu X., Yan F. (2018). Identifying medical diagnoses and treatable diseases by image-based deep learning. Cell.

[44] Pereira S., Meier R., Alves V., Reyes M., Silva C.A. (2018). Automatic brain tumor grading from MRI data using convolutional neural networks and quality assessment. Proceedings of the International Workshop on Machine Learning in Clinical Neuroimaging.

[45] Sayres R., Taly A., Rahimy E., Blumer K., Coz D., Hammel N., Krause J., Narayanaswamy A., Rastegar Z., Wu D. (2019). Using a deep learning algorithm and integrated gradients explanation to assist grading for diabetic retinopathy. Ophthalmology.

[46] Alexander B.M., Cloughesy T.F. (2017). Adult glioblastoma. J. Clin. Oncol..

[47] Van Tellingen O., Yetkin-Arik B., De Gooijer M., Wesseling P., Wurdinger T., De Vries H. (2015). Overcoming the blood–Brain tumor barrier for effective glioblastoma treatment. Drug Resist. Updat..

[48] Parillo M., Quattrocchi C.C. (2024). Brain tumor reporting and data system (BT-RADS) for the surveillance of adult-type diffuse gliomas after surgery. Surgeries.

[49] Li X., Morgan P.S., Ashburner J., Smith J., Rorden C. (2016). The first step for neuroimaging data analysis: DICOM to NIfTI conversion. J. Neurosci. Methods.

[50] Jenkinson M., Beckmann C.F., Behrens T.E., Woolrich M.W., Smith S.M. (2012). FSL. NeuroImage.

[51] Rohlfing T., Zahr N.M., Sullivan E.V., Pfefferbaum A. (2010). The SRI24 multichannel atlas of normal adult human brain structure. Hum. Brain Mapp..

[52] Avants B.B., Epstein C.L., Grossman M., Gee J.C. (2008). Symmetric diffeomorphic image registration with cross-correlation: Evaluating automated labeling of elderly and neurodegenerative brain. Med. Image Anal..

[53] Achanta R., Shaji A., Smith K., Lucchi A., Fua P., Süsstrunk S. (2012). SLIC superpixels compared to state-of-the-art superpixel methods. IEEE Trans. Pattern Anal. Mach. Intell..

[54] He K., Zhang X., Ren S., Sun J. Deep residual learning for image recognition. Proceedings of the IEEE Conference on Computer Vision and Pattern Recognition.

