# Supplementary material for: PHSP-Net: Personalized Habitat-Aware Deep Learning for Multi-Center Glioblastoma Survival Prediction Using Multiparametric MRI
Source: Bioengineering (Basel). 2025 Sep 15;12(9):978. doi: 10.3390/bioengineering12090978 (PMC12467337; doi:10.3390/bioengineering12090978)
Supplement: Supplementary file 1 [file bioengineering-12-00978-s001.zip › bioengineering-3846681-supplementary.pdf]

Supplementary Materials

1.Data Description and Preprocessing

After rigorous cohort curation, the UPENN-GBM and UCSF-PDGM datasets were randomly partitioned into a training set and an internal validation set at a 7:3 ratio. The LUMIERE and BraTS2021-TCGA-GBM cohorts served as two independent external test sets. The internal validation set remained locked throughout the study and was never used for hyperparameter tuning; it was employed exclusively for unbiased early stopping and model selection. For each of the four centres, we summarized key clinical variables—including patient age (mean ± std), sex, and overall survival (median)—and full demographic details are provided in **Supplementary Table S1**. Detailed results of the statistical tests for clinical characteristics are provided in **Supplementary Table S2**.

All images were acquired on 1.5 T or 3 T scanners from multiple vendors. DICOM files of the T1WI, T2WI, T1CE, and FLAIR modalities were converted to NIfTI format using dcm2niix[1]. Background regions were either cropped or zero-padded to yield a uniform matrix size of 155 × 240 × 240 voxels. Skull stripping was performed with the Brain Extraction Tool (BET) from the FMRIB Software Library (FSL)[2]. The skull-stripped T1CE volume was then non-linearly registered to the SRI24 atlas[3]. Subsequently, T1WI, T2WI, and FLAIR images were rigidly aligned to the previously registered T1CE using ANTs[4]. After resampling, all images had an isotropic spatial resolution of 1 mm × 1 mm × 1 mm. Intensities were clipped at the 1st and 99th percentiles and then z-score normalized within each modality.

Supplementary Table S1. Patient Characteristics of the Centers.

		UCSF-PDGM	UPENN-GBM	TCGA-GBM	LUMIERE
Age		59.65 ± 13.56	63.48 ± 11.88	59.27 ± 13.38	63.14 ± 9.72
Sex	Male	188	350	74	35
	Female	124	235	43	35
OS	Less than 1 year	115	265	57	20
	More than 1 year	198	320	60	50
	Media(Months)	15.22	12.82	12.32	17.62

Note. – The age of the patients is the mean value ± standard deviation.

Supplementary Table S2. Statistical tests for clinical characteristics of patients from different centers.

P-Value	UCSF-PDGM vs UPENN-GBM	UCSF-PDGM vs TCGA-GBM	UCSF-PDGM vs LUMIERE	UPENN-GBM Vs TCGA-GBM	UPENN-GBM vs LUMIERE	TCGA-GBM vs LUMIERE
Age	<0.001	0.803	0.042	<0.001	0.822	0.038
Sex	0.926	0.282	>0.999	0.041	>0.999	0.208
OS	0.141	0.055	0.653	0.444	0.233	0.041

Note. – Chi-square test was used for Sex, and independent samples t-test was used for Age and OS.

2.Radiomic Feature Extraction

The MRI sequences used in this study include T1WI, T2WI, T1CE, and FLAIR. The radiomic features were extracted using the PyRadiomics[5] library in a Python 3.9 environment, running on an Intel(R) Core(TM) i9-14900KF CPU. We modified the settings file to extract both original image features from the region of interest (ROI) as well as transformed image features. In total, 19 different image transformations were applied, including square image, square root image, logarithmic image, exponential image, filtered images with σ values of 1.0, 1.5, 2.0, 2.5, and 3.0, wavelet transformation (with sub-bands LLL, LLH, LHL, LHH, HLL, HLH, HHL, and HHH), and three-dimensional Local Binary Pattern (LBP) with levels = 2. Each transformed image included 18 first-order statistical features and 75 texture features. Therefore, for each transformation, 93 imaging features were extracted. In addition to these, the original image contributed 14 shape features, resulting in a total of 107 features from the original images. A detailed description of these features can be found in **Supplementary Table S3**.

It is important to note that we considered two types of ROI. One involved segmenting the tumor into three sub-regions: necrosis, enhancement, and edema. The other approach considered the entire tumor as a single ROI. When the ROI is the entire tumor, the number of features that can be extracted from one MRI modality for each patient is: (107 + 93 x 19) x 4 = 7496: When the ROI is divided into three sub-regions (necrotic core, contrast-enhanced tumor, and peritumoral edema), the total number of features extracted from one MRI modality for each patient is: (107 + 93 x 19) x 4 x 3 = 22488. When constructing a machine learning model for OS prediction using the whole tumor features, we refer to this as the radiomics method. When constructing the model using the sub-region features, we refer to this as the habitat radiomics method.

Supplementary Table S3. List of radiomic features.

**Shape Features**	Elongation, Flatness, Least Axis Length, Major Axis Length, Maximum 2D Diameter Column, Maximum 2D Diameter Row, Maximum 2D Diameter Slice, Maximum 3D Diameter, Mesh Volume, Minor Axis Length, Sphericity, Surface Area, Surface Volume Ratio, Voxel Volume
**First-Order Features**	10 th Percentile, 90 th Percentile, Energy, Entropy, Interquartile Range, Kurtosis, Maximum, Mean Absolute Deviation, Mean, Median, Minimum, Range, Robust Mean Absolute Deviation, Root Mean Squared, Skewness, Total Energy, Uniformity, Variance
**Texture Features**	**GLCM:** Autocorrelation, Cluster Prominence, Cluster Shade, Cluster Tendency, Contrast, Correlation, Difference Average, Difference Entropy, Difference Variance, Id, ldm, Idmn, Idn, Imc1, Imc2, Inverse Variance, Joint Average, Joint Energy, Joint Entropy, MCC, Maximum Probability, Sum Average, Sum Entropy, Sum Squares<br>**GLDM:** Dependence Entropy, Dependence Non Uniformity, Dependence Non Uniformity Normalized, Dependence Variance, Gray Level Non Uniformity, Gray Level Variance, High Gray Level Run Emphasis, Large Dependence Emphasis, Large Dependence High Gray Level Emphasis, Large Dependence Low Gray Level Emphasis, Low Gray Level Emphasis, Small Dependence Emphasis, Small Dependence High Gray Level Emphasis, Small Dependence Low Gray Level Emphasis<br>**GLRLM:** Gray Level Non Uniformity, Gray Level Non Uniformity Normalized, Gray Level Variance, High Gray Level Run Emphasis, Long Run Emphasis, Long Run High Gray Level Emphasis, Long Run Low Gray Level Emphasis, Low Gray Level Run Emphasis, Run Entropy, Run Length Non Uniformity, Run Length Non Uniformity Normalized, Run Percentage, Run Variance, Short Run Emphasis, Short Run High Gray Level Emphasis, Short Run Low Gray Level Emphasis<br>**GLSZM:** Gray Level Non Uniformity, Gray Level Non Uniformity Normalized, Gray Level Variance, High Gray Level Zone Emphasis, Large Area Emphasis, Large Area High Gray Level Emphasis, Large Area Low Gray Level Emphasis, Low Gray Level Zone Emphasis, Size Zone Non Uniformity, Size Zone Non Uniformity Normalized, Small Area Emphasis, Small Area High Gray Level Emphasis, Small Area Low Gray Level Emphasis, Zone Entropy, Zone Percentage, Zone Variance<br>**NGTDM:** Busyness, Coarseness, Complexity, Contrast, Strength

GLCM gray level co-occurrence matrix, GLDM = Gray Level Dependence Matrix, GLRLM = Gray Level Run Length Matrix, GLSZM = Gray Level Size Zone Matrix, NGTDM = Neighboring Gray Tone Difference Matrix.

3. Radiomic Features with Non-zero Coefficients Selected by LASSO

To construct the machine learning models for overall survival (OS) prediction, we employed the Least Absolute Shrinkage and Selection Operator (LASSO)[6] as an effective feature selection method. By introducing an L1 regularization term, LASSO not only helps prevent overfitting but also identifies the most relevant imaging features that contribute significantly to OS prediction.

The non-zero coefficients selected during the LASSO process correspond to features that exhibit substantial predictive power for survival outcomes. By learning from these selected features, the model is better able to capture imaging-derived patterns associated with patient prognosis.

LASSO is a regularized linear regression technique that integrates an L1 penalty into its loss function. The penalty encourages sparsity by shrinking less important feature coefficients to zero, thus enabling automatic feature selection while controlling model complexity.

The penalty coefficient λ plays a critical role in this process. A larger λ enforces stronger regularization, potentially reducing more coefficients to zero, while a smaller λ allows more features to be retained in the model. Proper tuning of λ is essential to strike a balance between underfitting and overfitting, ultimately optimizing model performance.

The coefficient path plot, often visualized during the LASSO procedure, depicts how feature coefficients evolve as λ varies. Features whose coefficients remain non-zero over a wide range of λ values are considered robust and clinically relevant, as they consistently contribute to OS prediction. These paths provide insights into the stability and importance of specific radiomic features across the regularization spectrum.

Supplementary Figure S1. LASSO Selects Features with Non-zero Coefficients

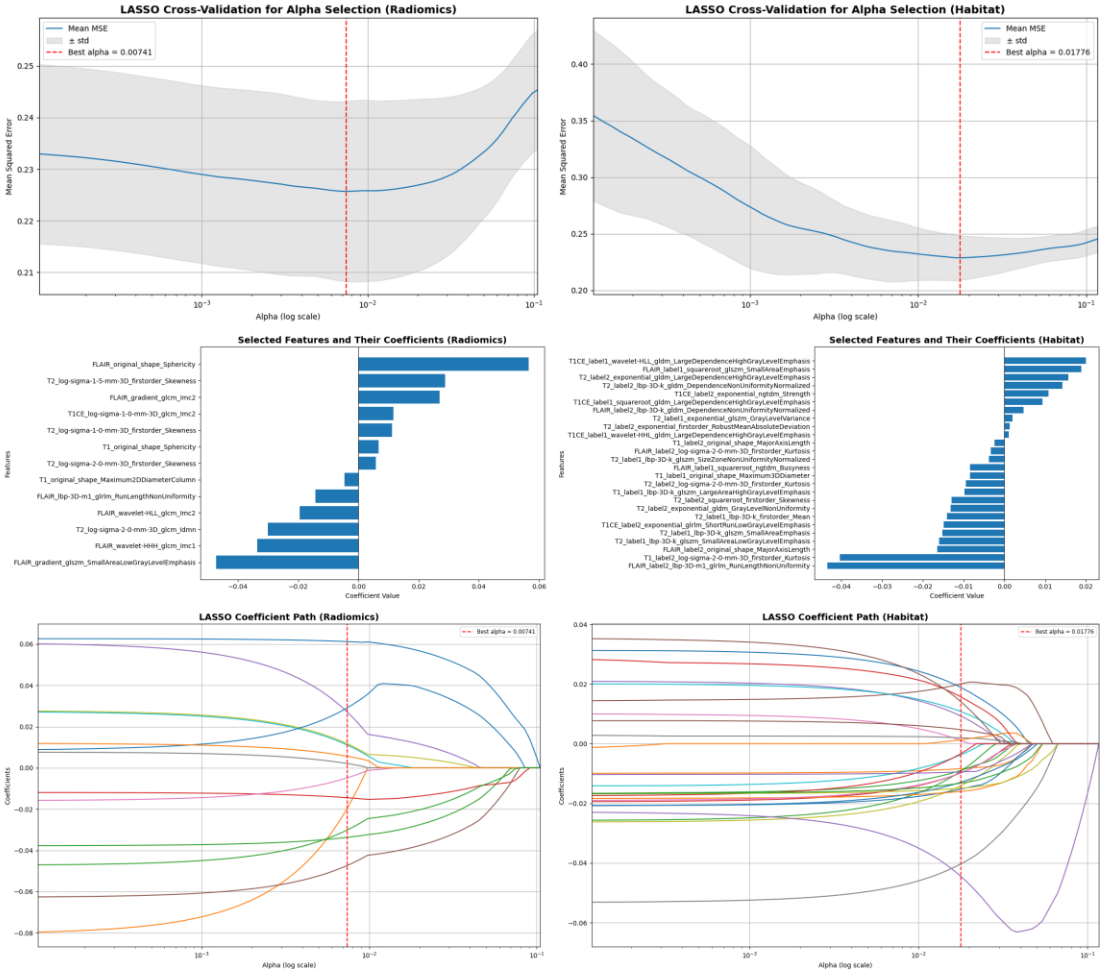

Note. "label1" and "label2" represent the necrotic tumor core and edema area, respectively.

4. Description of the Deep Learning and Traditional Machine Learning Models Used

After the LASSO (Least Absolute Shrinkage and Selection Operator) selected the non-zero coefficients in the training set, we used these features to build a Support Vector Machine (SVM) model for predicting overall survival (OS) as a binary classification task. The threshold for classifying patients into long survival (OS ≥ 12 months) and short survival (OS < 12 months) was set at 12 months.

SVM is a widely used supervised learning model for both classification and regression tasks. The core idea behind SVM is to find a hyperplane that best separates the samples of different classes. In a 2D space, this hyperplane is a line; in 3D space, it is a plane; and in higher-dimensional spaces, it is a hyperplane.

While SVM is a common machine learning model, it is not the focus of this study, so further details will not be discussed here. Radiomics typically treats the tumor as a homogeneous region and extracts features across the entire tumor, ignoring the inherent heterogeneity of glioblastoma multiforme (GBM). Habitat radiomics, on the other hand, considers the heterogeneity of GBM and often divides the tumor into regions through voxel clustering, aiming to extract more detailed imaging features, thereby improving disease understanding and prediction accuracy. However, habitat radiomics usually requires the tumor to be divided into sub-regions before feature extraction. The accuracy of predictions depends heavily on the precision of the sub-region delineation, and there is also the issue of individual variability—whether the number of sub-regions should be the same across all patients is still a matter of discussion.

In response to the challenges associated with traditional habitat radiomics, we propose a Personalized Habitat-Aware Survival Prediction Network (PHSP-Net) for personalized end-to-end habitat segmentation and accurate OS prediction in GBM patients. Specifically, we first apply Simple Linear Iterative Clustering (SLIC)[7] on the four modality MRI scans of each patient's tumor. This clustering is performed on the basis of the tumor being segmented into three main regions: necrosis, enhancement, and edema. Within each of these sub-regions, the tumor is re-clustered into 24 smaller sub-regions (habitats). SLIC is a superpixel segmentation algorithm that simplifies the k-means clustering process, creating compact, nearly uniform-sized superpixels with good shape regularity and compactness.

After the initial habitat segmentation, the data is fed into the PHSP-Net network for training. The network starts with a 3x3x3 convolution layer, followed by batch normalization and ReLU activation, to increase the channel number of the feature maps and better capture features related to OS prediction. The network then includes three consecutive Adaptive Habitat Encoding and Fusion Modules (AHEFM). This module consists of three layers of dilated convolutions and habitat attention mechanisms. The dilated convolutions progressively expand the receptive field of the kernel to capture finer variations in the image while maintaining high-resolution feature maps (i.e., ensuring the input and output image sizes remain consistent and are not reduced by convolution operations). The habitat attention mechanism is designed to refine the model's understanding of habitat segmentation. Specifically, the mean pixel value for each sub-region is computed in the feature map after the first convolution operation, followed by Sigmoid normalization to obtain the habitat attention weights. These weights are then applied to the convolutional output, performing pixel-wise multiplication to apply attention correction to the habitat segmentation. This attention mechanism can help to merge similar habitats, allowing for more personalized habitat segmentation. Additionally, skip connections[8] are incorporated to build a deeper network and prevent gradient

explosion.

After passing through the initial 3x3x3 convolution and three AHEFM modules, the network produces 64 high-resolution feature maps. Unlike traditional Class Activation Mapping (CAM) techniques, which extract the feature maps from the last convolution layer via gradients after model training, we use 1x1x1 convolutions to replace the traditional fully connected layers, reducing the 64 high-resolution feature maps to a single channel. These maps are then globally average-pooled to identify the regions the model focuses on. A Sigmoid activation is applied to predict the binary OS classification result. Through end-to-end learning, the model selects the most relevant feature map for OS prediction as the final visual output. This simple yet efficient convolutional neural network (CNN) allows us to predict OS accurately while providing a high-resolution visual explanation for model decision-making, which has the potential to aid in patient management and clinical decision-making.

5. PHSP-Net Detailed Implementation Process

$$F_0 = \text{Relu}(\text{BN}(\text{Conv3D}_{3\,x\,3\,x\,3}(x))) \tag{1}$$

Formula (1) represents the initial convolution layer operation, where a 3×3×3 convolution is applied to enhance the feature map's channel depth, thereby improving the model's training capacity. BN refers to the batch normalization layer, and ReLU denotes the non-linear activation function.

$$M = \text{I}(\sum_{r=1}^{R} M_r > 0) \tag{2}$$

$$F_r = F \odot M_r \tag{3}$$

$$F_r = \frac{\sum_{d,h,w} F_r}{\sum_{d,h,w} M_r + \epsilon} \tag{4}$$

$$w_r = \delta[W_2\left(\text{ReLU}(W_1(F_r))\right)] \tag{5}$$

$$w_r = \max(w_r, \alpha) \tag{6}$$

$$A = \sum_{r=1}^{R} M_r \odot w_r \tag{7}$$

$$F' = F \odot A \odot M \tag{8}$$

The **Habitat Attention Mechanism** is described in **Formulas (2) to (8)**. I(x) is an indicator function that sets the value to 1 if x is greater than 0, and to 0 otherwise. Mr represents the habitat mask, and R denotes the maximum number of sub-regions. In our study, R=72. In **Formula (3)**, Fr refers to the feature map for a particular habitat region, and F is the feature map for the entire image. **Formula (4)** is the global average pooling operation for each habitat, which computes the average value of the pixels within a habitat. ϵ is a very small constant to prevent division by zero. In **Formula (5)**, W1 and W2 represent 1×1×1 convolution operations, with W1 used to reduce the number of channels to 14 of the original, and W2 used to restore the channels back to the original size. α is a constant set to 0.5, which prevents the habitat pixel values from becoming zero or excessively small. Through **Formula (6)**, we calculate the attention for each habitat, and **Formula (7)** aggregates the attention across all habitats. F′ is the feature map for the habitat after attention is applied.

$$F_1 = H[\text{ReLU}(\text{BN}(\text{Conv3D}_{3\,x\,3\,x\,3}^{d=1}(F_{in})))] \tag{9}$$

$$F_2 = H[\text{ReLU}(\text{BN}(\text{Conv3D}_{3\,x\,3\,x\,3}^{d=2}(F_1)))] \tag{10}$$

$$F_3 = H[\text{ReLU}(\text{BN}(\text{Conv3D}_{3\,x\,3\,x\,3}^{d=3}(F_2)))] \tag{11}$$

$$F_{out} = \text{ReLU}(F_3 + \text{Shortcut}(F_{in})) \tag{12}$$

The full operation of an AHEFM module includes Formulas (9) to (12). It involves three consecutive dilated convolutions with progressively increasing dilation rates, where *d* denotes the dilation rate, *H* denotes the habitat attention mechanism, and *Shortcut* denotes the residual connection.

$$F_{end} = \mathrm{Conv3D}_{1\times1\times1}\left(\mathrm{F}_{\mathrm{out}_3}\right) \tag{13}$$

After processing through three AHEFM modules, the feature map is visualized using a 1×1×1 convolution to reduce the channel count to 1. The final visualization feature map Fend is the output from the end-to-end deep learning network.

$$\mathrm{y}' = \delta(GAP(F_{end})) \tag{14}$$

$$\mathcal{L} = -\mathrm{y} \cdot \log(y') - (1 - y) \cdot \log(1 - y') \tag{15}$$

Once the visualization feature map is obtained, the GBM patient overall survival prediction is performed using Formulas (14) and (15). The global average pooling operation is applied to the final output feature map to compute the logits. Then, the Sigmoid function is applied to obtain the predicted value of the model. The loss function is the Binary Cross Entropy Loss (BCELoss).

6.Model Performance Testing

The confusion matrix is a specific table format used to describe the performance of classification models. It provides a clear way to evaluate the classifier's performance by comparing the predicted results with the actual labels.

		Prediction	
		**Positive**	**Negative**
**Reference**	**Positive**	**True Positive**	**False Negative**
	**Negative**	**False Positive**	**True Negative**

True Positive (TP): A sample is truly positive, and the model correctly identifies it as positive.

False Negative (FN): A sample is truly positive, but the model incorrectly classifies it as negative.

False Positive (FP): A sample is truly negative, but the model incorrectly classifies it as positive.

True Negative (TN): A sample is truly negative, and the model correctly identifies it as negative.

$$Accuracy = \frac{TP + TN}{\mathrm{TP} + TN + FP + FN} \tag{1}$$

Accuracy represents the proportion of all test samples that are correctly classified.

$$Recall\ (Sensitivity) = \frac{TP}{\mathrm{TP} + TN} \tag{2}$$

Recall indicates the proportion of actual positive samples that are correctly identified.

$$Specificity = \frac{TN}{TN + FP} \tag{3}$$

Specificity reflects the proportion of actual negative samples that are correctly identified as negative.

$$Precision = \frac{TP}{TP + FP} \tag{4}$$

Precision describes the proportion of truly positive cases among all samples predicted as

positive.

$$F1\ Score = 2 \cdot \frac{Precision \cdot Recall}{Precision + Recall} \tag{5}$$

F1 Score is the harmonic mean of precision and recall, providing a measure of the model's balance between the two.

Supplementary Figure S2. Confusion matrix results for different models on the validation and test sets.

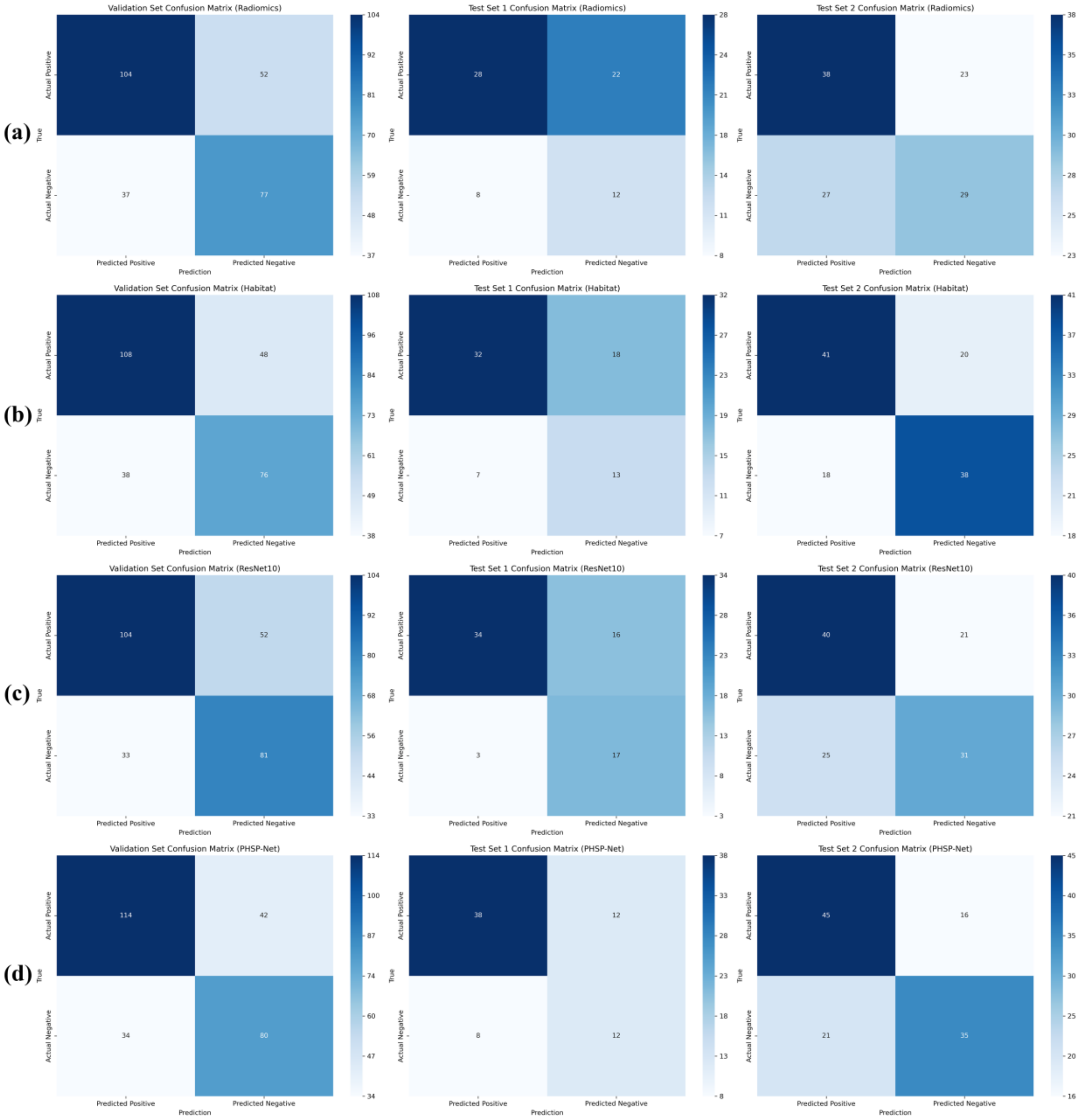

Supplementary Table S4. DeLong test results for AUROC comparison among models.

	Model	PHSP-Net	ResNet10	Habitat Radiomics	Radiomics
Validation	PHSP-Net		< 0.001	0.003	< 0.001
	ResNet10	< 0.001		0.034	< 0.001
	Habitat Radiomics	0.003	0.034		< 0.001
	Radiomics	< 0.001	< 0.001	< 0.001	
Test1	PHSP-Net		0.014	0.019	< 0.001
	ResNet10	0.014		0.029	0.037
	Habitat Radiomics	0.019	0.029		0.023
	Radiomics	< 0.001	0.037	0.023	
Test2	PHSP-Net		0.011	0.021	< 0.001
	ResNet10	0.011		0.025	0.007
	Habitat Radiomics	0.021	0.025		0.016
	Radiomics	< 0.001	0.007	0.016	

Supplementary Figure S3. Bar charts of objective evaluation metrics for each model on the validation set and test sets.

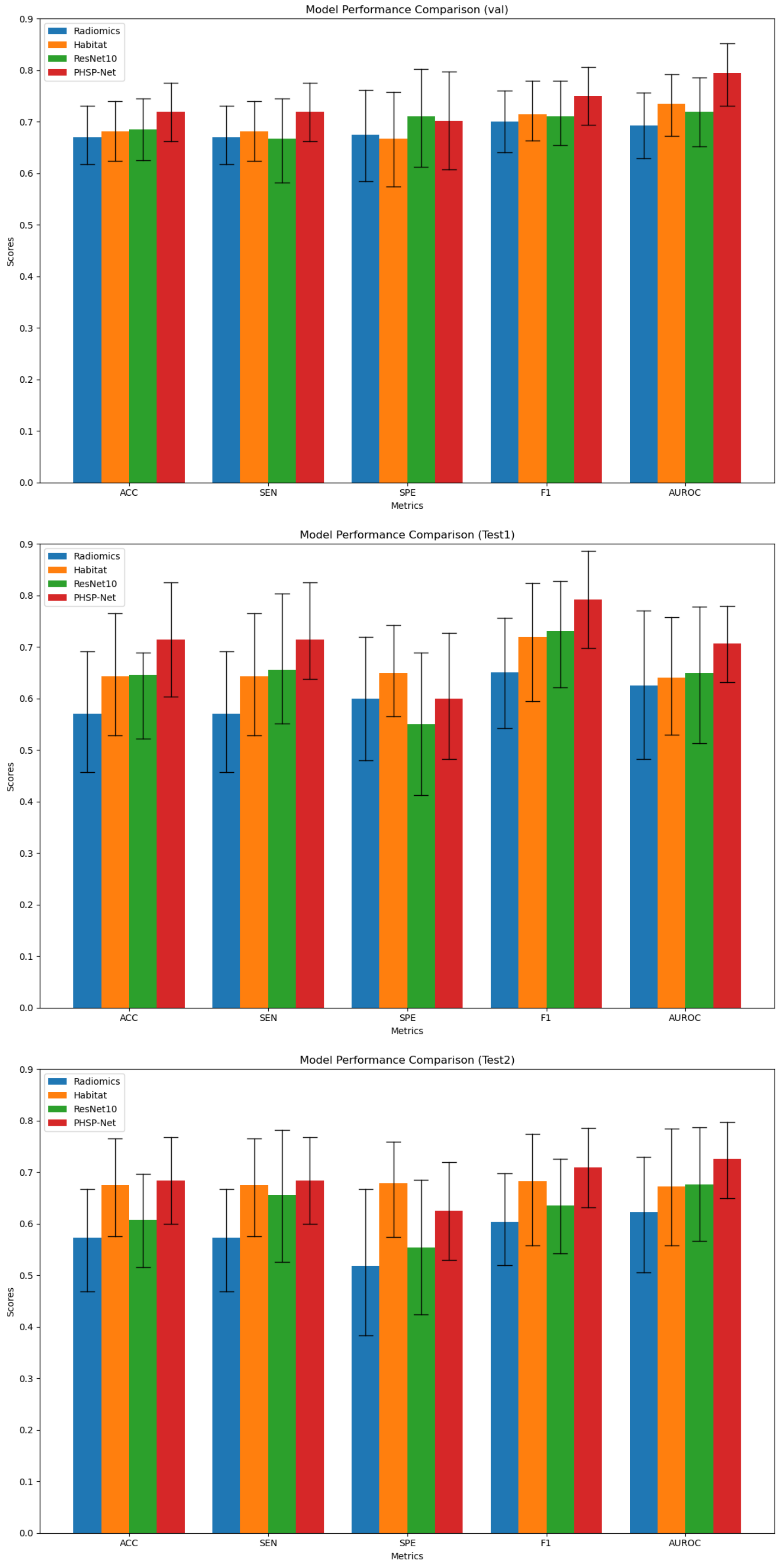

Reference

[1]. Li X, Morgan PS, Ashburner J, Smith J, Rorden C. The first step for neuroimaging data analysis: DICOM to NIfTI conversion[J]. Journal of neuroscience methods. 2016,264:47-56.

[2]. Jenkinson M, Beckmann CF, Behrens TE, Woolrich MW, Smith SM. Fsl[J]. Neuroimage. 2012,62(2):782-90.

[3]. Rohlfing T, Zahr NM, Sullivan EV, Pfefferbaum A. The SRI24 multichannel atlas of normal adult human brain structure[J]. Human brain mapping. 2010,31(5):798-819.

[4]. Avants BB, Epstein CL, Grossman M, Gee JC. Symmetric diffeomorphic image registration with cross-correlation: evaluating automated labeling of elderly and neurodegenerative brain[J]. Medical image analysis. 2008,12(1):26-41.

[5]. Lambin P, Rios-Velazquez E, Leijenaar R, Carvalho S, Van Stiphout RG, Granton P, et al. Radiomics: extracting more information from medical images using advanced feature analysis[J]. European journal of cancer. 2012,48(4):441-6.

[6]. Tibshirani R. Regression shrinkage and selection via the lasso[J]. Journal of the Royal Statistical Society Series B: Statistical Methodology. 1996,58(1):267-88.

[7]. Achanta R, Shaji A, Smith K, Lucchi A, Fua P, Süsstrunk S. SLIC superpixels compared to state-of-the-art superpixel methods[J]. IEEE transactions on pattern analysis and machine intelligence. 2012,34(11):2274-82.

[8]. He K, Zhang X, Ren S, Sun J, editors. Deep residual learning for image recognition. Proceedings of the IEEE conference on computer vision and pattern recognition; 2016.
